# A mutation in the *POT1* gene is responsible for cardiac angiosarcoma in *TP53*-negative Li–Fraumeni-like families

**DOI:** 10.1038/ncomms9383

**Published:** 2015-09-25

**Authors:** Oriol Calvete, Paula Martinez, Pablo Garcia-Pavia, Carlos Benitez-Buelga, Beatriz Paumard-Hernández, Victoria Fernandez, Fernando Dominguez, Clara Salas, Nuria Romero-Laorden, Jesus Garcia-Donas, Jaime Carrillo, Rosario Perona, Juan Carlos Triviño, Raquel Andrés, Juana María Cano, Bárbara Rivera, Luis Alonso-Pulpon, Fernando Setien, Manel Esteller, Sandra Rodriguez-Perales, Gaelle Bougeard, Tierry Frebourg, Miguel Urioste, Maria A. Blasco, Javier Benítez

**Affiliations:** 1 Human Genetics Group, Spanish National Cancer Research Center (CNIO), Melchor Fernandez Almagro 3, Madrid 28029, Spain; 2 Center for Biomedical Network Research on Rare Diseases (CIBERER), Madrid 28029, Spain; 3 Telomeres and Telomerase Group, Spanish National Cancer Research Center (CNIO), Madrid 28029, Spain; 4 Department of Cardiology. Hospital Universitario Puerta de Hierro, Mahadahonda, Madrid 28222, Spain; 5 Department of Cardiovascular Development and Repair, Centro Nacional de Investigaciones Cardiovasculares (CNIC), Madrid 28029, Spain; 6 Department of Pathology. Hospital Universitario Puerta de Hierro Majadahonda, Madrid 28222, Spain; 7 Oncology Department, Clara Campal Comprehensive Cancer Center, Sanchinarro, Madrid 28050, Spain; 8 Department of Experimental Models of Human Disease. Instituto Investigaciones Biomédicas (CSIC/UAM), Madrid 28029, Spain; 9 Bioinformatic Unit, Sistemas Genómicos, Paterna 46980, Spain; 10 Medical Oncology Service, Hospital Universitario Lozano Blesa, Zaragoza 50009, Spain; 11 Medical Oncology Service, Hospital General de Ciudad Real, Ciudad Real 13005, Spain; 12 Familial Cancer Clinical Unit, Spanish National Cancer Research Center (CNIO), Madrid 28029, Spain; 13 Cancer Epigenetics and Biology Program (PEBC), Bellvitge Biomedical Research Institute (IDIBELL), Barcelona 08908, Spain; 14 Department of Physiological Sciences II, School of Medicine, University of Barcelona, Barcelona 08007, Spain; 15 Institució Catalana de Recerca i Estudis Avançats (ICREA), Barcelona 08010, Spain; 16 Cytogenetics Unit, Spanish National Cancer Research Center (CNIO), Madrid 28029, Spain; 17 Genetics Department, Rouen University Hospital, Rouen 76000, France

## Abstract

Cardiac angiosarcoma (CAS) is a rare malignant tumour whose genetic basis is unknown. Here we show, by whole-exome sequencing of a *TP53*-negative Li–Fraumeni-like (LFL) family including CAS cases, that a missense variant (p.R117C) in *POT1* (*protection of telomeres 1*) gene is responsible for CAS. The same gene alteration is found in two other LFL families with CAS, supporting the causal effect of the identified mutation. We extend the analysis to *TP53*-negative LFL families with no CAS and find the same mutation in a breast AS family. The mutation is recently found once in 121,324 studied alleles in ExAC server but it is not described in any other database or found in 1,520 Spanish controls. *In silico* structural analysis suggests how the mutation disrupts POT1 structure. Functional and *in vitro* studies demonstrate that carriers of the mutation show reduced telomere-bound POT1 levels, abnormally long telomeres and increased telomere fragility.

## Abstract (web summary)

Genetic factors that cause cardiac angiosarcoma are unknown. Calvete *et al*. show that a missense mutation in *protection of telomeres* 1 (POT1) gene causes cardiac angiosarcoma by affecting the POT1 function and, consequently, telomere length and stability.

## Graphical abstract

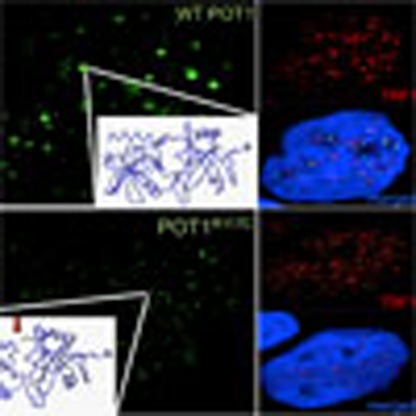

The incidence of primary cardiac tumours is estimated to be between 0.001 and 0.003% in general population^1^. Cardiac angiosarcoma (CAS) is a very rare malignant tumour that represents <10% of cardiac malignancies Patients are generally diagnosed at advanced stages with very poor prognosis and short survivals^2^. Patients with sporadic AS had a 5-year survival rate of 14% while familial cancer patients had a mean survival time of 4 months. Age of familial CAS onset shows a wide range but most patients affected are younger than 65 years of age^2^. Information about familial CAS is very scarce and as far as we now, only two families have been reported presenting both an earlier age of onset than sporadic^3^,^4^ No genes responsible for these cases have been identified so far, thus humpering early diagnosis and effective treatment, which involves surgical excision combined with chemotherapy, and heart transplantation only for patients with no evidence for metastasis.

On the other hand, Li–Fraumeni syndrome (LFS) is characterized by the presence of different types of tumours including sarcomas and ASs in multiple locations such as liver, spleen, breast, head and neck, and rarely heart^5^. In most cases, LFS is triggered by the mutations in the *TP53* gene (>80%), which is the causal gene that confers susceptibility to the development of different tumours. In addition, Li–Fraumeni-like (LFL) families have similar clinical presentation and family characteristics. However, they are generally diagnosed at a later age of onset and rarely associated with mutations in *TP53* (<20% of cases)^6^,^7^.

Here we describe the identification by whole-exome sequencing of *POT1* (*protection of telomeres* 1) gene as responsible of *TP53*-negative LFL families with CAS, as well as other tumour types.

POT1 is a component of the so-called shelterin complex (POT1, TRF1, TRF2, TIN2, TPP1 and RAP1) that binds to telomeres and has fundamental roles in chromosome stability and regulation of telomerase activity at chromosome ends^8^,^9^.

POT1 possesses two N-terminal OB domains, which confer to this protein high specificity for single-stranded DNA sequence 5′-TAGGGTTAG-3′, thereby binding to the telomeric G-strand overhang^10^,^11^,^12^. In addition, POT1 binds through its C terminus to TPP1 and anchors POT1 to the shelterin complex at telomeres^13^,^14^,^15^. POT1 regulates telomere length (TL) by preventing telomerase access to telomeres by sequestering the DNA terminus^12^,^16^,^17^. POT1 loss-of-function mutations have been related with telomere lengthening and chromosomal instability^18^,^19^. Recently, germline mutations in the *POT1* gene were found in familial melanoma and glioma tumours^20^,^21^,^22^, as well as in somatic chronic lymphocytic leukemia (CLL)^23^.

Here we identify a new missense variant in *POT1* (p.R117C) as responsible of LFL locus specifically associated with CAS and demonstrate that mutation carriers show reduced telomere-bound POT1 levels, abnormally long telomeres and increased telomere fragility, highlighting a new role of *POT1* as a high susceptibility gene in familial cancer and opening therapeutical opportunities for prognosis and treatment in families with CAS.

## Results

### Whole-exome sequencing (WES) and candidate variant studies

**Figure 1:**
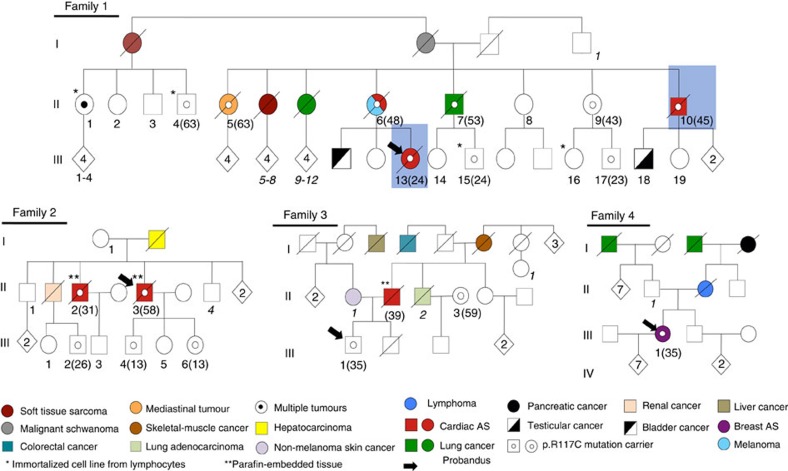
Pedigrees of *TP53*-negative LFL families. Families 1, 2 and 3 have three, two and one members with cardiac angiosarcomas (CAS), respectively. CAS and other tumours are shown (colour code below the Fig.). Exomes of blue-squared CAS cases were sequenced. One member of family 4 presented with breast angiosarcoma. Only family members of whom DNA samples were available are numbered (1–19) in bold and italics. Age of onset (CAS cases) and age of sample (mutation carriers) is shown in brackets. Age of onset of other tumours is shown in the Table 1. ‘*’ indicates immortalized lymphocyte cell lines from family 1: II-1 and II-4 (64- and 65-years old, respectively) and III-15 and III-16 (34- and 35-years old, respectively). ‘**’ indicates paraffin-embedded tumour tissue available. Black arrows show the probandus for each family.

A *TP53*-negative LFL Spanish family with three CAS cases and eight members with different tumours across generations were ascertained from the Cancer Genetic Consultancy of CNIO, (Fig. 1 and Table 1). Two members affected by CAS (II-10 and III-13) were selected for WES (Fig. 1; Methods for details). About 85% of target regions were observed with coverage >20 × . After filtering according to our homemade pipeline and GATK-based variant calling (Methods), we only found 394 variants in common between both samples, which were used for downstream filtering. Ten variants fulfilled our prioritization criteria, taking forward annotation and putative damaging effects into account (Supplementary Table 1). After Sanger validation and segregation studies in other members of this family, variants annotated in *POT1* (Chr7:124503601G>A), *SPEG* (chr2:220355477) and *EIF2AK* (chr2:37347280) were validated. We decided to further study *POT1*, as other germline and somatic mutations of this gene were recently found in familial melanoma and glioma tumours^20^,^21^,^22^, as well as in CLL^23^, respectively. *POT1* variant found in both CAS members (p.R117C) was highly deleterious and was neither described in the Exome variant server, Ensembl, dbSNP, 1000 genomes and COSMIC databases nor present in melanoma and glioma families with *POT1* mutations^20^,^21^,^22^. The variant was recently found once (heterozygous) in 121,324 studied alleles (frequency: 8.2 × 10^−6^) in ExAC server. p.R117C mutation was not found in 1,520 Spanish control individuals.

We could study the complete *POT1* gene in two additional Spanish LFL families with CAS (Fig. 1, families 2 and 3) and strikingly, we found the same variant p.R117C. Then, we extended the study of the POT1 gene to 19 probands belonging to *TP53*-negative LFL families, with no CAS. Nine of them had developed different AS types and 10 presented with sarcomas (Table 1). We found one Spanish family with breast AS and other tumours carrying the same mutation.

We assessed the founder effect of the mutation, by performing haplotype studies with seven single nucleotide polymorphisms (SNPs) covering the gene (Methods). Six different haplotypes were found from the analysis of control Spanish families. Only families 1 and 2 had enough members for this study and both presented the most frequent haplotype (42%) (Supplementary Table 2).

### *In silico* studies

**Figure 2:**
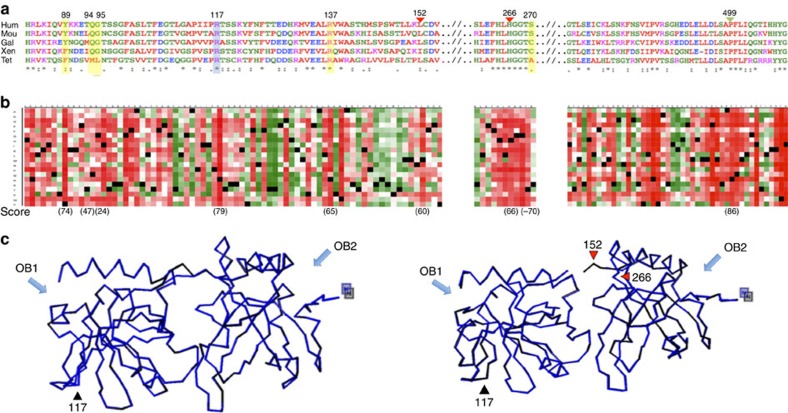
*In silico* studies. (**a**) Amino acid conservation across representative phylogeny of POT1 orthologues (tet, *Tetraodon nigroviridis*; xen, *Xenopus laevis*; gal, *Gallus gallus*; hum, *Homo sapiens*; mou, *Mus musculus*). The mouse POT1a gene sequence was used in the alignment. Position p.117 is shown in blue; mutations described in previous studies are shown in yellow. Triangles indicate positions with putative conformational changes (red) and loss of TPP1-binding site (green) due to the p.R117C mutation. ‘*’ indicates amino acid (aa) conserved in all POT1 orthologues. (**b**) Heat map representation shows the tolerance to independent aa substitutions (*y*-axis) for each position of the protein (*x*-axis). Dark red indicates the highest score for a deleterious effect (score 100); white indicates a small effect; green indicates a neutral effect/no effect (score −100); and black represents the corresponding wild-type residue. Deleteriousness effect score is shown for highlighted positions. (**c**) Putative tertiary structure. p.152 and p.266 residues change PACC score value driving a putative protein conformation change. Left: homology-based three-dimensional model of human POT1 (Uniprot, Q9NUX5). Right: structural impact of the p.R117C mutation using the same algorithm from Protein Model Portal (Uniprot, PSI_SBKB). Black triangle shows the loop where p.117 is located. Red triangles show the principal detected structural changes (p.152 and p.266). Blue arrows show OB1 and OB2 domains.

The p.R117 position is highly conserved across selected representative species that suggest the same residue for the last common ancestor within the phylogeny (Fig. 2a). p.R117C mutation was located within the OB1/OB2 domains of the protein, similarly to the germline mutations found in familial melanoma and glioma^20^,^21^,^22^ (Table 2). Tolerance to independent amino acid (aa) substitutions was calculated in a heat map representation using PredictProtein^24^ showing a highly deleterious effect (Fig. 2b).

Position p.117 was found to be part of a short linear peptide motif of a disordered/unstructured region based on previous predictions^10^ (hot loops), which have a critical conformation^25^ and are related to alterations in predicted accessibility and protein flexibility. Thus, the PACC (predicted solvent accessibility in square Angstroem) was calculated for the residues of POT1^R117C^ protein model that were described to be located within the OB fold (from p.142 to p.152) and with stacking interaction to DNA (p.31, p.62, p.89, p.161, p223, p.245, p.266 and p.271) in POT1 protein according to the study by Lei *et al*.^10^ Two residues (p.152 and p.266) had significantly different PACC scores in POT1^R117C^ protein model (Supplementary Table 3). p.152 and p.266 are oriented closed together and located at the border of domains OB1 and OB2. The consequence of this accessibility change predicts a disruption of the interaction between OB1 and OB2 in a putative three-dimensional model of the POT1^R117C^ protein (Fig. 2c).

To complete the study of these putative conformational changes observed in POT1^R117C^, PACC scores for the same residues were calculated for predicted protein models encompassing previously described mutations in the OB1 domain in melanoma and glioma tumours (p.Y89C and p.Q94E^20^, p.G95C^22^, and p.R137H^21^). Moreover, we also included a protein model with a deletion from p.1 to p.126 residues (POT1^ΔOB1^) as described by Loayza and De Lange^12^ and a second protein model encompassing a deletion of the whole OB1 domain (cd04497: hPOT1_OB1_like (E-value: 3.43e-45); interval from p.10 to p.141). Both p.152 and p.266 PACC score changes observed in POT1^R117C^ were not found in the rest of the studied protein models (Supplementary Table 3).

Finally, we searched for the putative protein–protein-binding regions of wild-type (wt) POT1 protein and 14 positions were found to be significantly relevant (threshold>20) (Supplementary Table 4). The same positions were studied in all mutated protein models. A putative protein-binding region annotated in position p.499 was significantly lost in the POT1^R117C^ protein model (Supplementary Table 4). p.499 is a well-conserved position with high deleterious tolerance to aa change (86,4) at the conserved POT1 C terminus within the TPP1-binding domain (Fig. 2b)^13^. Interestingly, this protein-binding region was lost in the protein model with the deletion of the whole OB1 domain (from p.10 to p.141) but was unaffected in the other mutant models and POT1^ΔOB1^ (with a deletion in OB1 domain from p.1 to p.126) (ref. 12).

### Expression and protein studies

**Figure 3:**
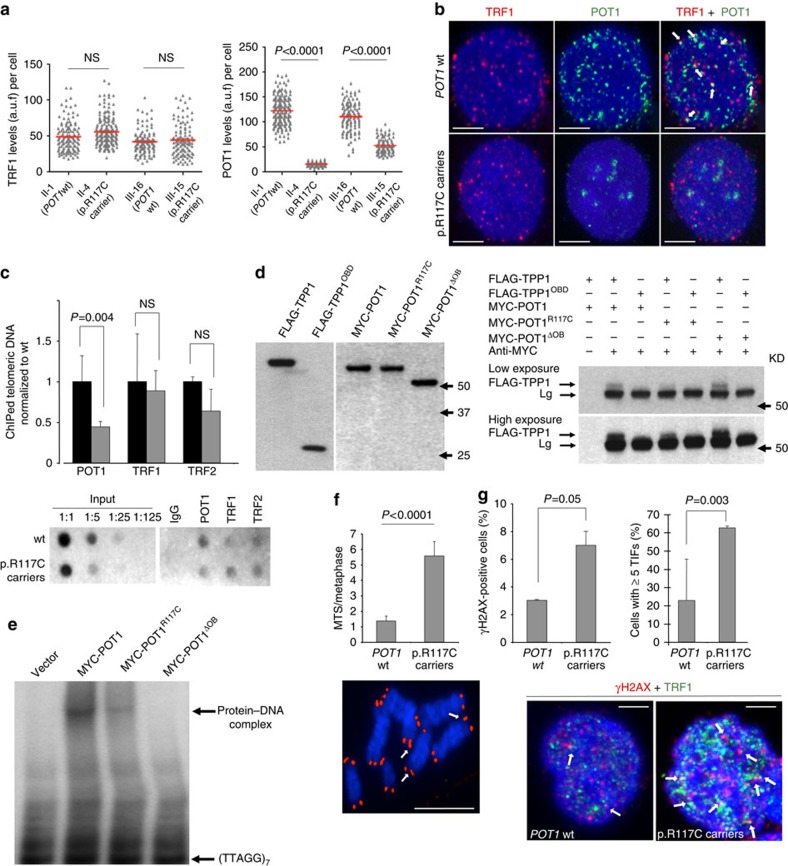
*POT1*^*R117C*^ mutation affects telomere binding and induces telomeric damage. (**a**) Quantification of telomere-bound TRF1 (left panel) and POT1 (right panel) protein levels in immortalized LCLs corresponding to p.R117C carriers and non-carriers. Two independent experiments with replicated samples were performed. a.u.f., arbitrary units of fluorescence. (**b**) Representative images of TRF1 (red) and POT1 (green) double immunofluorescence in wild-type and p.R117C carriers. White arrows indicate colocalization of both proteins (yellow spots). Scale bar, 5 μm. (**c**) Quantification of telomeric DNA bound to POT1, TRF1 and TRF2 by ChIP analysis. IgG was used as negative control. Results were normalized to input chromatin. Black bars, wild-type; grey bars, p.R117C carriers. Two independent experiments from each genotype were performed. Lower panel: representative ChIP dot-blot is shown. (**d**) Left panel: western blot analysis of *in vitro*-translated FLAG-TPP1, FLAG-TPP1^OBD^, MYC-POT1, MYC-POT1^R117C^ and MYC-POT1^ΔOB1^. Right Panel: p.R117C substitution decreased POT1 binding capacity to TPP1. Co-immunoprecipitation assays of the *in vitro*-translated proteins. FLAG-TPP1 was pulled down with MYC antibody and revealed by FLAG antibody. FLAG-TPP1 lacking the TIN2 and POT1 binding domain, FLAG-TPP1OBD, and a POT1 mutant lacking its OB1 domain were used as controls. Two exposures are showed. (**e**) p.R117C substitution decreased POT1 binding capacity to telomeric ssDNA. Electrophoretic mobility shift assay of [^32^P]-labelled oligonucleotide (5′-TTAGGG-3′)_7_ in the presence of the indicated *in vitro*-translated POT1 proteins. Data from two independent experiments are shown in **d**,**e**. (**f**) Upper panel: quantification of multitelomeric signal (MTS) events per metaphase in primary lymphocytes by telomeric FISH (*n*=2 in triplicate). Lower panel: example of MTS (white arrows). Red fluorescence shows telomere signals. Scale bar, 5 μm. (**g**) Quantification of cells positive for γH2AX (left) and per cent of cells with >5 telomeric induced foci (TIFs) (right) in immortalized LCLs (*n*=2 in triplicate). Lower panel: representative images of γH2AX and TRF1 immunofluorescence. White arrows show examples of TIFs (yellow spots) with anti-TRF1 (green fluorescence) and anti-γH2AX (red fluorescence). Scale bar, 5 μm. Values are expressed as mean+s.e. The two-tailed student’s unpaired *t*-test was used for the statistical analysis, NS, not significant. DAPI (blue) was used for DNA labelling.

To study whether the missense variant (p.R117C) affects POT1 transcriptional and protein levels, we performed quantitative PCR (qPCR) from complementary DNA (cDNA) and western blot (WB) analyses from primary lymphocytes (PL) from carriers and in non-carriers. We found no significant differences in *POT1* mRNA levels in PL from carriers compared with non-carriers (Supplementary Fig. 1). Similarly, we did not find significant differences in POT1 protein levels (two-tailed student’s *t*-test, *P*=0.1) as determined by WB (Supplementary Fig. 1). Next, we set to determine POT1 levels localized at telomeres by double immunofluorescence with POT1 and TRF1 antibodies (another telomere-binding protein from the shelterin complex used here to localize telomeres)^26^. To this end, we used immortalized lymphoblastoid cell lines (LCLs) derived from members II-1, II-4, III-15 and III-16 (Fig. 1, family 1). Immunofluorescence levels with anti-TRF1 antibody were similar for all family members independently of the carrier status (Fig. 3a,b). In contrast, the intensity of POT1 telomeric foci was significantly lower in p.R117C carriers (two-tailed student’s *t*-test, *P*<0.0001) than controls and there was also a decrease in the number of co-localizations (Fig. 3a,b).

To address the effect of this mutation on the binding of POT1 to telomeric chromatin *in vivo*, we performed chromatin immunoprecipitation (ChiP) with the four LCLs using antibodies against POT1, TRF1 and TRF2 (Methods). The hybridization signal of telomeric DNA immunoprecipitated with anti-TRF1 and anti-TRF2 (positive telomeric controls) was not significantly different in mutation carriers compared with non-carriers (Fig. 3c). However, a significant reduction of hybridization signal was observed in mutation carriers when telomeric DNA was immunoprecipitated with anti-POT1 antibodies (two-tailed student’s *t*-test, *P*=0.004).

POT1 is recruited to telomere through interaction with TPP1 (ref. 13). The reduced levels of telomere-bound POT1 observed in mutation carriers (Fig. 3a–c), as well as the *in silico* analysis (Supplementary Table 4) suggest that POT1^R117C^ might be defective in TPP1 binding. To test this, we performed co-immunoprecipitation assays with *in vitro*-translated MYC-tagged POT1^R117C^ and FLAG-tagged TPP1. As controls, we used MYC-tagged wt POT1 and a mutant MYC-POT1^ΔOB1^ that has been shown to be defective in single strand DNA binding but functional in its interaction with TPP1 (ref. 12). In addition, we also used a FLAG-tagged mutant TPP1^OBD^ that lacks both the POT1 and TIN2 interaction domain^27^. Equal amounts of the different MYC-POT1 and FLAG-TPP1 variants were mixed as indicated and TPP1-POT1 complexes immunoprecipitated with anti-MYC antibody (Fig. 3d; Supplementary Fig. 2). As expected, FLAG-TPP1 was pulled down with MYC-POT1 and MYC-POT1^ΔOB1^ while FLAG-TPP1^OBD^ was not detected with any of the *POT1* variants used in the assay. However, immunoprecipitation of MYC-POT1^R117C^ recovered FLAG-TPP1 to a much lesser extent than wt MYC-POT1. Indeed, FLAG-TPP1 was only faintly detected when the immunoblots were developed under long exposure time (Fig. 3d). These results confirmed that mutant POT1^R117C^ is affected in its ability to bind TPP1.

We next sought to determine whether the p.R117C substitution affected the ability of POT1 to bind to the 3′ end of the G-rich telomeric overhang, as suggested by the *in silico* analysis. *In vitro*-translated MYC-POT1, MYC-POT1^ΔOB1^ and MYC-POT1^R117C^ were incubated with radio-labelled telomeric single-stranded DNA (ssDNA) and with c-MYC (9E10) antibody. Visualization of the protein-DNA complexes by electrophoretic mobility shift assay confirmed that wt POT1 efficiently bound telomeric ssDNA, whereas POT1^R117C^ bound telomeric ssDNA less efficiently (Fig. 3e; Supplementary Fig. 2). As expected, POT1^ΔOB1^ was unable to bind to the telomeric sequence.

### Studies in telomeres

In line with dysfunctional POT1 that is usually associated with increased DNA damage at telomeres (the so-called telomere-induced foci (TIF)) and occurrence of telomere fragility, we observed significantly increased multitelomeric signal events (MTS) in mutation carriers compared with non-carriers (two-tailed student’s *t*-test, *P*<0.0001) (Fig. 3f) and an increase of positive cells for γH2AX (two-tailed student’s *t*-test, *P*=0.05) a DNA damage marker (Fig. 3g). Double immunofluorescence staining with γH2AX and TRF1 antibodies was performed to determine TIFs. The results showed a significant increase in the percentage of cells with more than five TIFs in carriers as compared with non-carriers (two-tailed student’s *t*-test, *P*=0.003) (Fig. 3g). Of note, we did not observe end-to end fusions in mutation carriers.

**Figure 4:**
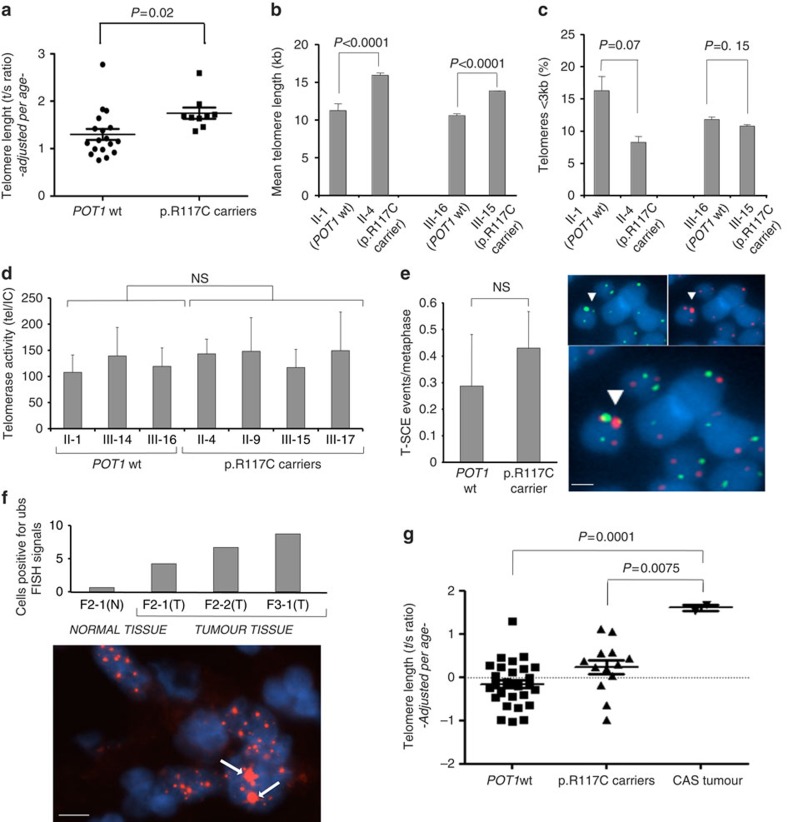
POT1^R117C^ carriers present longer telomeres. (**a**) Telomere length (TL) analysis by qPCR (*t*/*s* ratio) for family 1 members (*n*=29). (**b**) TL analysis by qFISH of primary immortalized LCLs corresponding to non-carriers (II-1 and III-16) and p.R117C carriers (II-4 and III-15). (**c**) Percentage of telomeres shorter than 3 kb of primary immortalized LCLs corresponding to non-carriers (II-1 and III-16) and p.R117C carriers (II-4 and III-15). (**d**) TRAP Tel/IC ratio values for telomerase activity calculated from primary lymphocytes of different members of family 1 (*n*=7). In **b**–**d** two independent experiments with samples in triplicate were performed. (**e**) Left panel: number of telomere sister chromatid exchange (T-SCE) events/metaphase in primary lymphocytes by CO-FISH (*n*=2 individuals per genotype in triplicate). Right panels: representative CO-FISH images showing the leading (green) and lagging (red) telomere strands. T-SCEs are indicated with arrows. DAPI (blue) was used for DNA labelling. Below a magnified merge image is shown. Scale bar, 1 μm. (**f**) Upper panel: per cent of cells positive for ultra-bright spots (ubs) at telomeres by FISH in three different paraffin-embedded cardiac tumour (T) and normal (N) tissue samples carrying the mutation from members of family 2 (F2) and 3 (F3). Lower panel: examples of large red spots corresponding to positive signals (white arrows). DAPI (blue) was used for DNA labelling. Scale bar, 10 μm. (**g**) TL adjusted for age of wt and p.R117C carriers of all members of families 1, 2 and 3 and the 3 CAS tumours. DNA from CAS samples was extracted from paraffin-embedded tissues. Values are expressed as mean+s.e. and the two-tailed student’s unpaired *t*-test was used for the statistical analysis, NS, not significant.

We evaluated the effect of the p.R117C mutation in TL. We measured it by qPCR in peripheral blood lymphocytes from members of family 1. TL values were adjusted for age. We consistently found longer telomeres in mutation carriers compared with non-carriers (two-tailed student’s *t*-test, *P*=0.02) (Fig. 4a). TL was also calculated with fluorescence *in situ* hybridization quantitative detection (quantitative fluorescence *in situ* hybridization (qFISH))^28^ and we validated that TL was significantly longer in mutation carriers compared with controls (two-tailed student’s *t*-test, *P*<0.0001) (Fig. 4b). In addition, a higher percentage of short telomeres <3 kb was found in non-carriers compared with p.R117C mutant carriers comparing older members (II-1 and II-4, 64.5 old in average) (Fig. 4c). Otherwise, no significant differences among members of family 1 were found regarding *in vitro* telomeric repeat amplification (TRAP) telomerase activity (Fig. 4d).

Abnormal telomeres elongation could be the consequence of increased telomere recombination, to the so-called alternative lengthening of telomeres (ALT). We evaluated this possibility by quantification of recombination events specifically at telomeric repeats, the so-called telomere sister chromatid exchanges (T-SCEs). We observed a similar frequency of T-SCE in mutations carriers (0.43±0.14) compared with wt controls (0.29±0.19) (Fig. 4e) ruling out a possible ALT mechanism of telomere elongation in lymphocytes.

Finally, paraffin-embedded tissues were also studied. Loss of heterozygosity of *POT1* locus was evaluated in four CAS tumours from families 1 and 2. Loss of heterozygosity was observed in only one of the studied cases while the other three cases presented a heterozygosis profile. The three CAS tumours from families 2 and 3 (one also with normal cardiac tissue) were also studied for ultra-bright spots (ubs) in telomeres. FISH for ubs in telomeres showed an increased number of ubs-positive signals (5.56 positive signals × 100/total signals on average) compared with normal tissue (0.68 positive signals × 100/total signals; Fig. 4f). This alteration correlated with the TL of tumours, which was highly increased compared with wt samples (*P*=0.0001) and mutation carriers (*P*=0.0075) adjusted for age (two-tailed student’s *t*-test) (Fig. 4g).

### *In vitro* experiments

**Figure 5:**
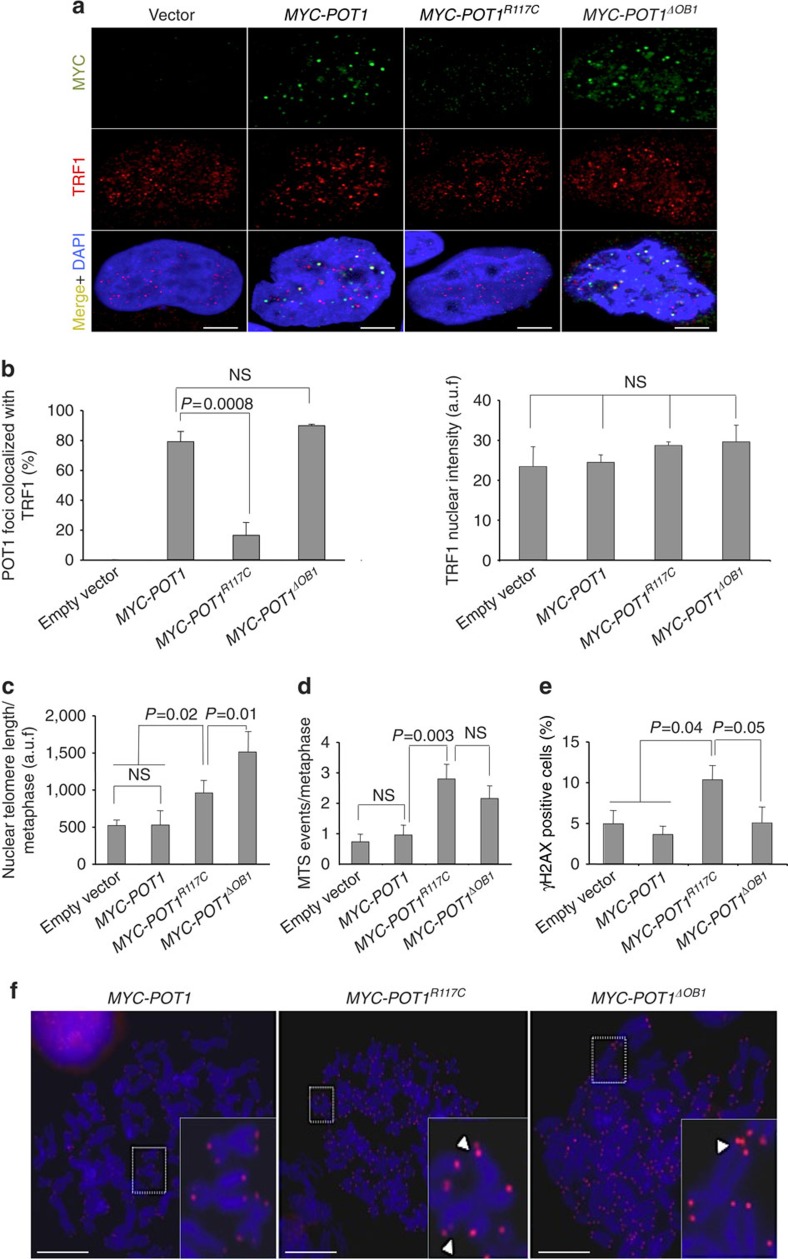
Heterologous POT1^R117C^ expression induces telomere lengthening. (**a**) Representative images of MYC (green) and TRF1 (red) double immunofluorescence in Hela cells with endogenous expression of POT1 protein infected with retroviral empty vector, *MYC-POT1, MYC-POT1*^*R117C*^ and *MYC-POT1*^ΔOB1^. DAPI (blue) was used to DNA labelling. Scale bar, 5 μm. (**b**) Quantification of MYC-POT1 and TRF1 colocalization foci (left panel) and of nuclear TRF1 foci intensity (right panel) (a.u.f., arbitrary units of fluorescence) in Hela cells infected with retroviral empty vector, *MYC-POT1, MYC-POT1*^*R117C*^ and *MYC-POT1*^ΔOB1^. (**c**,**d**) Quantification of nuclear telomere length per metaphase (a.u.f) (**c**) and of multitelomeric signal (MTS) events/metaphase (**d**) in Hela cells infected with retroviral empty vector, *MYC-POT1, MYC-POT1*^*R117C*^ and *MYC-POT1*^*ΔOB1*^. (**e**) Per cent of γH2AX-positive cells in Hela cells infected with retroviral empty vector, *MYC-POT1, MYC-POT1*^*R117C*^ and *MYC-POT1*^*ΔOB1*^. (**f**) Representative qFISH images of metaphase spreads. Examples of MTS are shown in the magnified insets (arrow). Red fluorescence shows telomere signal. DAPI (blue) was used for DNA labelling. Scale bar, 5 μm. Two independent infections were performed. Values are expressed as mean+s.e. The two-tailed student’s unpaired *t*-test was used for the statistical analysis, NS, not significant.

As the identified mutation is present in heterozygosis in all the analysed carriers, we hypothesize that POT1^R117C^ protein functions in a dominant-negative manner. To further validate this possibility, we create heterologous Hela cell lines using retroviral vectors to express wt *MYC-POT1*, *MYC-POT1*^*R117C*^ and *MYC-POT*^Δ*OB1*^ alleles^26^ in the presence of endogenous POT1. Double immunofluorescence microscopy analysis with MYC and TRF1 antibodies of Hela cells showed that wt and mutant Myc-POT1^ΔOB1^ proteins but no cells expressing the MYC epitope alone (empty vector) show colocalization of both telomeric proteins MYC-POT1 and TRF1. Hela cells expressing MYC-POT1^R117C^ showed a similar pattern than those expressing the vector alone and only few cells presented few MYC foci that colocalized with TRF1 (Fig. 5a,b). Quantification of TRF1 nuclear intensity revealed no significant differences among Hela cells expressing either the empty vector or the wt and mutant *POT1* alleles (Fig. 5a,b). Decreased amount of telomere bound of MYC-POT1^R117C^ protein in Hela cells is in agreement with the decreased amount of protein observed in lymphocytes by immunofluorescence and ChIP analysis using POT1 antibodies (Fig. 3a–c).

To address, whether heterologously *MYC-POT1*^*R117C*^ overexpression recapitulates the observed effects in lymphocytes we analysed the functional effects of POT1^R117C^ expression in Hela cells. TL in Hela cells infected with retroviral empty vector, *MYC-POT1, MYC-POT1*^*R117C*^ and *MYC-POT1*^Δ*OB1*^ was measured by quantitative telomere FISH on metaphase spreads. Increased TL was observed in both Hela cells expressing *MYC-POT1*^Δ*OB1*^ and *MYC-POT1*^*R117C*^ compared with both cells infected with empty vector and *MYC-POT1* after 30 population doublings (Fig. 5c,f).

Finally, analysis of chromosome aberrations in metaphase spreads of the different transduced Hela cells, revealed a moderate but significant increase in MTS events in those expressing *MYC-POT1*^*R117C*^ and *MYC-POT1*^Δ*OB1*^ as compared with those expressing either the empty vector or wt *MYC-POT1* (Fig. 5d,f). In addition, an increase in γH2AX-positive cells was detected in Hela cells infected with *MYC-POT1*^*R117C*^ as compared with those harbouring either the empty vector, *MYC-POT1 wt* or *MYC-POT1*^Δ*OB1*^ (Fig. 5e).

## Discusion

We have found a novel damaging missense variant (p.R117C) in the *POT1* gene in *TP53*-negative LFL families with CAS and other tumours. Mutations in *POT1* were recently associated with familial melanoma^20^,^21^ and glioma^22^ and as a driver for CLL^23^, uncovering a new role of this gene not only as telomere protector, but also as one of the main responsible genes for development of different familial cancer types. The mutation was the same in the four studied families. Haplotype studies suggested a possible founder effect within the Spanish population although larger studies are necessary to confirm this issue.

There are several consequences of this mutation that we have demonstrated by *in silico*, *in vivo* and *in vitro* studies. Thus, *in silico* studies showed that the p.R117C substitution putatively disrupted the interaction between OB1 and OB2 (Fig. 2d) and affect the protein-binding site to TPP1 (Supplementary Table 4), which might result in the loss of capability of the POT1^R117C^ protein to interact with ssDNA and to be recruited to telomeres. The predicted conformation change might be explained as a consequence of the substitution of a polar basic aa (Arginine) with a non-polar thiol aa (Cysteine) at position p.117 with the capability to generate disulfide bonds.

*In silico* analyses were confirmed by *in vivo* assays analysing the binding of mutated POT1 protein to telomeres. Double immunofluorescence with POT1 and TRF1 in immortalized LCLs suggests that the variant has an important effect on the levels of POT1 at telomeres by showing significant decreased levels in mutation carriers. *In vitro* assays demonstrated that POT1^R117C^ is deficient at both, in TPP1 interaction and in telomeric ssDNA binding, affecting POT1 function.

Although the mutation was located within the OB1/OB2 domains of the protein, similarly to the germline mutations found in familial melanoma and glioma^20^,^21^,^22^ (Table 2), it was specific of CAS. In fact, only a member from family 1 carrying the mutation (II-6) developed melanoma, and no members from reported families with melanoma or gliomas carrying *POT1* mutations presented CAS^20^,^21^,^22^. The fourth family (Fig. 1, family 4) did not have any member with CAS but the family structure was small and we cannot rule out the possibility that asymptomatic carriers of the mutation will develop a CAS in the future.

These findings stress the importance of POT1 domains in cancer development and raise the intriguing open question about the correlation between the genotype and the phenotype. Our *in silico* studies suggest that the putative conformational changes in POT1 proteins due to the mutations described in melanoma and glioma tumours are different from those observed for POT1^R117C^ (Supplementary Tables 3 and 4). Because POT1 is described in tight coordination with the rest of shelterin complex proteins, we cannot rule out that different conformational changes might have different effects on the shelterin complex function.

Similarly to other members of the shelterin complex, disruption of POT1 function was associated to increased DNA damage at telomeres and occurrence of telomere fragility^23^. In our case, defective POT1^R117C^ function has shown to lead to a moderate increase in MTS, and in telomere DNA damage, that could account for the genomic instability that these cases present. Moreover, increased TL was confirmed in mutation carriers. This is in agreement with previously described POT1 mutations in CLL and familial melanoma and glioma patients that showed abnormally elongated telomeres^20^,^21^,^22^,^23^. In our cases, TL was markedly different between mutation carriers and non-carriers within older members, suggesting that there was a cumulative effect in telomere elongation with age (Fig. 4b,c). Differences observed in the amount of telomeres shorter than 3 Kb in older members suggest a downfall in the telomere shortening through aging, indicating abnormal telomere biology in mutation carriers. We showed similar *in vitro* TRAP telomerase activity levels in both carriers and non-carriers, indicating no defects in telomerase assembly or function (Fig. 4d). Thus, the longer telomeres observed in mutation carriers are rather due to alterations in *in vivo* telomerase recruitment and function on telomeres. In fact, POT1 has been shown to be important to prevent telomerase access to telomeres by sequestering the DNA terminus^12^,^16^,^17^. We show that POT1^R117C^ is defective in both binding to TPP1 and binding to ssDNA telomeric DNA. It is therefore tempting to speculate that POT1^R117C^ fails to inhibit telomerase due to the reduced telomere-bound POT1^R117C^, as well as to defective binding to telomeric G-strand overhang. In addition, given the defective binding of POT1^R117C^ to single G-strand overhang (Fig. 3d,e), telomeric damage could also stem from defective maintenance of the 3′ G-strand overhang needed for proper T-loop formation. Indeed, abnormal telomere elongation due to the consequence of increased telomere recombination (ALT) was ruled out as no differences in T-SCE were found in lymphocytes between carriers and non-carriers. Thus, lack of functional POT1 seems to affect telomere structure and expose them to abnormal elongation by a normal telomerase activity.

The telomeric phenotypes observed in lymphocytes from p.R117C variant carriers were validated by *in vitro* studies in Hela cell lines created using retroviral vectors expressing wt *MYC-POT1*, *MYC-POT1*^*R117C*^ and *MYC-POT1*^*ΔOB1*^ (ref. 26) in the presence of endogenous POT1. We confirmed that the POT1^R117C^ protein acts in a dominant-negative manner, analogous to that previously reported for the POT1^DOB1^ mutant^26^, as well as for others *POT1* variants found in CLL^23^. In this system, we also detected decreased POT1^R117C^ protein levels at telomeres, moderate increase in MTS incidence, higher telomeric DNA damage and a telomere lengthening effect. So, it is tempting to speculate that telomere elongation induced by this mutation in *POT1* may constitute the underlying molecular mechanism favouring tumour incidence.

The identification of *POT1* mutation in CAS is important, since it provides the possibility for earlier diagnosis of people at risk. Currently this tumour is diagnosed in advanced stages when distant metastases are present and the survival is very poor, as surgical resection is not effective. Larger studies are necessary to fully assess the clinical spectrum associated with this mutation and other mutations along the whole *POT1* gene. Future work is needed to address POT1^R117C^ relation not only with familial but also with sporadic CAS and its role in LFL cases including ASs other than cardiac.

## Methods

### Patients

Index cases from LFL families negative for mutations and large deletions of the *TP53* gene were selected for this study. Three of our families had members affected with CAS plus other tumours (Fig. 1). The rest of the families fulfilled the LFL criteria but they did not have any member with a CAS tumour (Table 1). Families were selected from the Spanish National Cancer Research Center (CNIO), Hospital Puerta de Hierro and Clara Campal Center (Spain), and Rouen University Hospital (France). DNA from most of the family members and whenever possible total mRNA and protein were collected from lymphocytes.

A group of 1,520 samples from a Spanish control population was used for genotyping the POT1 variant by denaturing high performance liquid chromatography^29^. Heteroduplex was amplified with primers of the exon 4, which encompassed the candidate variant (Supplementary Table 5). Genotyping was performed with WAVE Nucleic Acid Fragment System from Transgeomics and Navigator Software. Amplification was performed with standard conditions ((95 °C, 5 min; (44 °C, 30 seg.; 64 °C, 30 seg.; 72 °C, 45 seg.) × 30 cycle; 72 °C, 10 min.).

The study was approved by the ethics committee of the different centres and informed consent was obtained from all participants.

### Establishment of B LCLs

Lymphoblasts from different members of family 1 were immortalized: II-1 (aged 64), II-4 (aged 65), III-15 (aged 35) and III-16 (aged 34). LCLs were established by Epstein–Barr virus (EBV) transformation/infection of peripheral blood mononuclear cells (PBMC)^30^. PBMCs were incubated with B95-8 cell supernatant (containing EBV in the presence of 10 μg ml^−1^ of phytohemagglutinin in R.P.M.I1640 supplemented with 20% (v/v) heat-inactivated, foetal calf serum, 2 mM of L-Glutamine, 100 U ml^−1^ of penicillin, 100 μg ml^−1^ of streptomycin and 0.5 μg ml^−1^ of Amphotericin B (Gibco, Invitrogen)). Cells were plated and incubated at 37 °C, 5% CO_2_ and routinely grown in R.P.M.I1640 culture medium supplemented with 10% heat-inactivated foetal calf serum, 2 mM of L-Glutamine, 100 U ml^−1^ of penicillin and 100 μg ml^−1^ of streptomycin.

### Paraffin-embedded tissue samples

Paraffin-embedded tissue samples of CAS members from families 2 and 3 were collected (Fig. 1). DNA was extracted using the DNeasy Blood & Tissue Kit (Cat. No. 69504, Qiagen) following the manufacturer’s instructions. Selected tumour tissue was evaluated a pathologist (C.S.).

### Whole-exome sequencing

Exomes from two members of family 1 with CAS (II-10 and III-13) were captured and enriched using the SureSelect Human All Exon Kit (78 Mb) (Agilent Technologies). Enriched samples were paired-end sequenced on an Illumina Genome Analyzer II sequencing platform, using two lanes per sample and generating 78-bp-long reads. Filtering of reads and variant calling were done with the Rubioseq software suite of parallelized pipelines^31^. PCR duplicates, overrepresented sequences and low quality reads were filtered with a modular set of analyses considering per base and per sequence quality scores (Fastqc >30) and N content.

BWA files of short reads were aligned with the genome of reference (GRCh37/hg19). GATK-based variant calling was performed for aligned reads considering DP (read depth) values of >30 and QD (quality by depth) scores for a variant confidence of >1.00. A MAF <3% of was considered. Variants were also filtered by position (non-synonymous, essential splice site, frame shift or gain/loss of stops). Their potential damaging effect was assessed using the VEP^32^ script software package (including Sift, Polyphen and Condel damage predictors) from Ensembl.

Filtered variants were prioritized for segregation studies when they were (i) previously described in other pathogenic processes (COSMIC database), (ii) annotated in genes with related functions or involved in tumourogenetic processes, and (iii) annotated in genes suggested to be related to cardiac function.

Prioritized variants from 10 candidate genes were validated by Sanger sequencing and segregation studies were performed among the different members of family 1. Candidate variants that were found in the three CAS cases and mediastinal tumour patients were selected. PCR was performed using standard conditions (95 °C, 5 min; (44 °C, 30 s.; * °C, 30 s.; 72 °C, 45 s) × 30 cycle; 72 °C, 10 min). *: °C annealing of the corresponding primer. Sequences of the primers are listed in Supplementary Table 5.

### POT1 gene study

Sanger sequencing of the candidate *POT1* variant was performed in 19 probands from LFL families negative for *TP53* mutations. The entire *POT1* gene was sequenced for cases negative for the candidate mutation. The primers used for Sanger sequencing of the 15 exons are listed in Supplementary Table 5. PCR was performed using standard conditions ((95T °C, 5 min; (44T °C, 30 s; *T °C, 30 s; 72 °C, 45 s) × 30 cycle; 72T °C, 10 min). *: T °C annealing of the corresponding primer.

### Haplotype study

A haplotype study was performed to confirm the possible founder effect of our variant. Seven SNPs covering the gene were used (Supplementary Table 2). Selected trios (parent and two offspring) from families 1 and 2 were studied to establish the haplotype carrying the mutation. Trios from 15 healthy Spanish families were used to determine the different haplotypes in the Spanish population and their frequencies. The primers used for Sanger sequencing of the seven SNPs are listed in Supplementary Table 5.

### Real-time qPCR

Expression studies for *POT1* gene genes was performed with Real-Time qPCR with cDNA obtained from reverse transcription of 1,200 ng of total RNA from PBMCs using the High Capacity cDNA Reverse Transcription Kit (Applied Biosystems #4368814). PCR was carried out with ∼25 ng μl^−1^ of cDNA and the POWER SYBR green PCR Master Mix (Applied Biosystems #4367659). Quantification was performed using Sequence Detection System 7900HT (Applied Biosystems). Expression levels were evaluated with the ΔΔ*C*_t_ method^33^ in triplicate and normalized to the expression levels of *GAPDH*, which was used as a standard. PCR was run with exon 10 forward primer and exon 11 reverse primer to avoid amplification of genomic DNA (Supplementary Table 5). PCR was performed using standard conditions (95T °C, 5 min; (44T °C, 30 seg.; 58T °C, 30 seg.; 72 °C, 45 seg.) × 30 cycle; 72T °C, 10 min).

### Western blotting

WB was performed with 50 μg of total protein isolated from lysed PBMCs. Protein expression levels of POT1 were determined using rabbit polyclonal anti-POT1 antibody and normalized to GAPDH levels using anti-GAPDH antibody (Supplementary Table 6). The hybridization signal was quantified using ImageJ 1.43 u software (W. Rasband, the National Institutes of Health). Protein expression was evaluated with the ΔΔ*C*_t_ method^33^.

### ChIP assay

ChIP assays were performed with LCLs from family 1. Cells in culture were cross-linked with formaldehyde (1%) during 15 min at room temperature. The cross linking reaction was stopped by addition of glycine (0.125 M) during 5 min. Cells were washed twice with cold PBS, collected by centrifugation and lysed in lysis buffer (1%SDS, 10 mM EDTA, 50 mM Tris-HCl pH 8.0, protease inhibitors (P8340, Sigma)). Protein/DNA extracts were sonicated and centrifuged at 14,000 r.p.m. for 15 min. Protein concentration was determined (DC protein assay, Bio-Rad), and chromatin from 200 μg total protein extract was used per immunoprecipitation with 4 μl of either anti-POT1, anti-TRF2 or anti-TRF1 antibody (Supplementary Table 6) and protein A/G PLUS-agarose beads (Santa Cruz Biotechnology, sc-2003)^34^. The immunoprecipitated DNA was transferred to a Hybond N^+^ membrane using a dot-blot apparatus. The membrane was then hybridized with a telomeric probe containing 1.6 kb of 5′-TTAGGG-3′ repeats. Quantification of the signal was performed with ImageQuant software (Molecular Dynamics). The amount of telomeric DNA after ChIP was normalized to the total input telomeric DNA.

### Bioinformatics tools to assess the *in silico* studies

Heat map representation of independent substitutions for each position of the protein and aa tolerance test was performed with PredictProtein^24^. Secondary structures (β-strands, α-helix and loops) of the putative POT1 model were based on REPROFSec prediction. The predictions of the annotation (minimum REPROFSEc score of 5) of conserved secondary structures and evolutionary profiles for POT1 carrying the p.R117C mutation were based on several original prediction methods (NORSnet, DISOPRED2, PROFbval and Ucon) implemented in PredictProtein^24^. Solvent accessibility notation (PACC) was annotated using the PROFAcc prediction algorithm^35^ also implemented in ProteinPredict^24^. Protein binding regions were found using the ISIS algorithm^36^ and SomeNA predictor method (also implemented in ProteinPredict^24^). Annotation of residues was based on crystallography studies of POT1 (ref. 10). A homology-based three-dimensional model of human POT1 was taken from Uniprot (Q9NUX5). Protein modelling for POT1 carrying the p.R117C mutation (PSI_SBKB) was performed using the Protein model portal (available at http://www.proteinmodelportal.org/).

### Real-time qPCR of TL

TL was measured by qPCR using DNA from blood samples. Quantification of TL (*t*/*s* ratio) was done through quantification of telomere repeat copy number (*t*) and the *36B4* reference gene (*s*)^37^. Telomere repeats were amplified with TEL primers (tel 1, 5′-GGTTTTTGAGGGTGAGGGTGAGGGTGAGGGTGAGGGT-3′; tel 2, 5′-TCCCGACTATCCCTATCCCTATCCCTATCCCTATCC-CTA-3′), and *36B4* gene was amplified with 36B4u (5′-CAGCAAGTGGGAAGGTGTAATCC-3′) and 36B4d (5′-CCCATTCTATCATCAACGGGTACAA-3′) primers^37^. The final TL value for each sample was determined under standard conditions in three independent measurements. Independent measurements had a correlation of r 0.80. The coefficient of variation was obtained for telomere (average 5%, range 1–50%) and *36B4* (average 1.7%, range 0.02–16%). TL values were adjusted for age by calculating a regression line with 330 DNA controls^38^. Each sample was adjusted for the difference between the observed TL and the predicted value using the regression line. Following this method we adjusted *t*/*s* values obtained by qPCR (*y*=−0.0174*x*+1.96). PCR was performed using standard conditions (see real-time qPCR section).

### High-throughput qFISH

Mononuclear cells (150,000) from PBMCs were plated in triplicate onto Poly-L-Lysine pre-coated Greiner 96-well plates for 4 h at 37 °C. Poly-L-Lysine was removed before cell addition (100,000 lymphocytes per well). When lymphocytes attached to the wells, cells were washed three times with PBS and then fixed during 1 h at room temperature with methanol/acetic acid (3:1 vol/vol). The plates were overnight dried at 37 °C. The cells were rehydrated in PBS during 15 min, fixed for 1 min in 4% formaldehyde, treated with pepsin (1 mg ml^−1^, pH 2.0) at 37 °C for 10 min, washed with PBS, fixed for 1 min in 4% formaldehyde, washed with PBS and dehydrated in a ethanol series (70, 90, 100%). The Cy‐3‐labelled (C_3_TA_2_)_3_ PNA probe (Perseptive Biosystems, Bedford, MA) was dissolved in a hybridization buffer containing 70% formamide/10 mM Tris pH 7.0/0.25% (w/v) blocking reagent (0.5 μg ml^−1^) (Dupont, Boston, MA). The hybridization mixture was placed onto the wells, followed by DNA denaturation (5 min, 85 °C). After hybridization (2 h, room temperature), slides were washed twice with 70% formamide/0.1% BSA/10 mM Tris pH 7.2 for 15 min, followed by three washes of 5 min in TBS-Tween 0.08% containing DAPI (4′,6‐diamidino‐2‐phenylindole). The cells were dehydrated in a ethanol series (70, 90, 100%) and air dried. Finally, Moviol mounting medium was added to the wells^28^. Wells were sealed (Alumaseal; Sigma-Aldrich) and stored at 4 °C in the dark. Samples were processed by High-throughput microscopy within 48 h after sample preparation by using the Opera-Acapella system (Perkin Elmer) for image acquisition and analysis^28^. TL values were calculated using individual telomere spots.

### Telomeric FISH on metaphase spreads

Primary mononuclear cells from peripheral blood (PBMCs) were stimulated with 7.5 ng ml^−1^ 12-O-tetradecanoylphorbol-13-acetate in R.P.M.I medium supplemented with FBS, penicillin-streptomycin, β-mercaptoethanol, sodium pyruvate, nonessential aa and L-glutamine. Colcemid was added at a concentration of 0.1 μg ml^−1^ during 12 h. Cells were then recovered, subjected to hypotonic shock and fixed in methanol/acetic acid (3:1). QFISH hybridization on metaphase spreads was performed as described above for HT-QFISH^39^. Metaphase spreads were captured on a Leitz Leica DMRB fluorescence microscope. At least 10 metaphase spreads per subject were analysed, and chromosomal aberrations were quantified and represented as frequency per metaphase.

### Measurement of T-SCE events by chromosome orientation FISH

Measurement of sister telomere recombination events (T-SCE) was performed using chromosome orientation FISH (CO-FISH). Mononuclear cells from peripheral blood (PBMCs) were stimulated as above and sub-cultured in the presence of BrdU (5′-bromo-2′-deoxyuridine; Sigma) at a final concentration of 1 × 10^−5^ M, and then allowed to replicate their DNA once at 37 °C for 24 h. Colcemid was added at a concentration of 0.1 μg ml^−1^ during the last 6 h. Cells were then recovered, subjected to hypotonic shock and fixed in methanol/acetic acid (3:1). CO-FISH was performed using first a telomeric (5′-CCCTAA-3′)_7_ PNA probe labelled with Cy3 and then a second telomeric (5′-TTAGGG-3′)_7_ PNA probe labelled with Alexa-488 (Supplementary Table 6)^40^. Metaphase spreads were captured on a fluorescence microscope (DMRB).

### Retroviral expression

The pLPC-human *MYC-POT1* and pLPC-human *MYC-POT1*^*ΔOB1*^ (deleted OB1 domain) plasmids were a gift of de Lange (Addgene plasmid 12387 and 13241)^12^. The POT1 variant encoding p.R117C change, *MYC-POT1*^*R117C*^, was generated by site-directed mutagenesis of the pLPC-human *MYC-POT1* using the QuickChange XL site-directed mutagenesis kit (Agilent Technologies) using oligonucleotides hPOT1-R117C-F (5′-gggagcccctatcatacctTgcacttcaagcaagtat-3′) and hPOT1-R117C-R (5′-atacttgcttgaagtgcAaggtatgataggggctccc-3′). Retroviruses were packaged in 293T cells (ATCC-CRL-3216) using pCL-Ampho packaging vector. Hela cells (ATCC-CCL-2) were seeded onto p-10 plates to 30% of confluency 24 h before infection. Three consecutive infections every 12 h were performed by adding 5 ml of viral supernatant. Cells were allowed to recover for 24 h in growth medium before undergoing selection with puromycin for 3 days. Cells then underwent serial passaging and collected at the indicated population doubling points.

### Inmunofluorescence staining techniques

Lymphoblastoid (this work) and Hela (ATCC-CCL-2) cells were plated onto Poly-L-Lysine pre-coated coverslips, treated for 5 min with Triton X-100 buffer^41^ for nuclear extraction, fixed for 10 min in 4% buffered formaldehyde, permeabilized with 0.2% PBS-Triton for 5 min and blocked with foetal bovine serum in PBS for 1 h. Samples were incubated o/n at 4 °C with the primary antibody at 1:250 dilution. TIFs were detected using rabbit polyclonal anti-TRF1 and a mouse monoclonal antibody raised against phospho-histone2 H2AX-Ser139 (γH2AX) (Supplementary Table 6). Telomeric POT1 and TRF1 foci were detected using a rabbit polyclonal anti-POT1 and a mouse monoclonal antibody anti-TRF1 (Supplementary Table 6). MYC-POT1 was detected using a mouse monoclonal c-MYC antibody. Slides were further incubated with 488-Alexa or 555-Alexa-labelled secondary antibodies (Supplementary Table 6). Slides were mounted in Vectashield with DAPI. Confocal microscopy was performed at room temperature with a laser-scanning microscope (TCS SP5; Leica) using a Plan Apo 63 Å-1.40 numerical aperture oil immersion objective (HCX; Leica). Maximal projection of Z-stack images generated using advanced fluorescence software (LAS) was analysed with the Definiens XD software package. The DAPI images were used to detect signals inside the nuclei.

### TRAP analysis

Telomerase activity was measured using protein extract from PBMCs cultured in RPMI supplemented with 20% FBS and phytohemagglutinin during 4–5 days. Telomerase activity was determined under recommended standard conditions of the TRAPEZE Telomerase Detection S7700 Kit (Millipore) for TRAP using radioisotopic detection. Telomerase activity was determined in each sample using 0.5, 0.25 and 0.125 μg of protein extract and normalized with the internal control included in the assay^38^.

### Ultra-bright telomere spots in paraffin-embedded tissue

Ultra-bright telomere spots were detected by FISH on paraffin-embedded tissue slides. The Histology FISH Accessory Kit (DAKO) was used following the manufacturer’s instructions. A telomere-specific PNA-Cy3-labelled probe (DAKO) was used for the detection of ubs^42^. Tissue images were captured using a CCD camera with focus motor (Photometrics SenSys camera) connected to a PC running the Cytovision image analysis system (Applied Imaging Ltd., UK) and Z-stack software.

### G-strand binding and co-immunoprecipitation assays

The TNT-coupled reticulocyte lysate kit (Promega) was used to *in vitro* synthesize MYC-POT1, MYC-POT1^R117C^, MYC-POT1^ΔOB1^, FLAG-TPP1 and FLAG-TPP1^OBD^ (refs 12, 27). For co-immunoprecipitation assays, 3 μl of either MYC-POT1, MYC-POT1^R117C^ or MYC-POT1^ΔOB1^ with either 3 μl of either FLAG-TPP1 or FLAG-TPP1^OBD^ translation reaction and incubated at 37 °C for 20 min. About 500 μl of NETN buffer (20 mM Tris pH 8.0, 100 mM NaCl, 1 mM EDTA, 0.5% NP-40) and 1 μg of anti-MYC (9E10) were added to each protein mix and incubated at 4 °C. The antibody complexes were pulled down with protein A/G agarose beads, washed four times with NETN and eluted with 40 μl 2 × SDS loading buffer. The eluted proteins (10 μl) were separated by SDS–PAGE, transferred to nitrocellulose membranes and probed with a monoclonal FLAG antibody.

DNA-binding assays were performed in 20 μl reaction mixtures, 5 μl of each translation reaction was incubated with 10 nM 5′-[^32^P]-labelled telomeric oligonucleotide containing seven TTAGGG repeats, 1 μg of the nonspecific competitor DNA poly (dI-dC) and 2 μl of anti c-MYC (9E10) (C-40, Santa Cruz Biotechnology) in binding buffer (25 mM HEPES-NaOH pH 7.5, 100 mM NaCl, 1 mM EDTA and 5% glycerol)^23^. Reactions were incubated for 10 min at room temperature, and protein-DNA complexes were analysed by electrophoresis on a 6% polyacrylamide Tris-borate EDTA gel run at 80 V for 3 h.

### Statistics

Significance of expression differences among individuals grouped according to genotype (wt versus mutation carriers) was evaluated with the non-parametric Kolmogorov–Smirnov test to determine normal distribution of values within the groups. Student’s *t*-test was used for comparison of normally distributed values among genotypes. Differences were considered to be significant when the exact *P* value was <0.05.

### Study approval

Written informed consent was received from participants prior to inclusion in the study.

## Additional information

**Acession codes.** Whole genome sequencing data have been deposited in ArrayExpress under accession code E-MTAB-3858.

**How to cite this article:** Calvete, O. *et al*. A mutation in the *POT1* gene is responsible for cardiac angiosarcoma in *TP53*-negative Li–Fraumeni-like families. *Nat. Commun.* 6:8383 doi: 10.1038/ncomms9383 (2015).

## Supplementary Material

Supplementary Information Supplementary Figures 1 and 2 and Supplementary Tables 1-6

## Figures and Tables

**Table 1:** Tumour types of different members of the studied families.

**p.R117C**	**Family**		**Tumour type**									
			**Sarcoma**				**Breast**	**CNS**	**Melanoma**	**Lung**	**Other**	**Multiple primary tumours** *
			**Angiosarcoma**	**Soft**	**Bone**	**NS**						
Present	1	Carriers	3 CAS (24,45,58)**	1 (69)		1 (93)			1 (48)	2 (47,61)		1 melanoma+CAS
		NT		1 (56)						1 (43)		
	2	Carriers	2 CAS (32,41)									
		NT									1 liver (54) 1 kidney (50)	
	3	Carriers	2 CAS (40)									
	4	Carriers	1 breast (32)								1 PTC (35)	1 BA+PTC
		NT									1 lymphoma (38)	
Not Present	5			3 (41,45,50)			1 (U)	2 astroc. (12,24)			1 NHL neurofibromas (24)	1 astroc. +NHL+CM
	6			1 (42)				1 astroc. (12)			1 CRC (40)	
	7				2 (45,49)						2 stomach (65,68), 2 CCR (27,71)	
	8			1 (2)			1 (44)		1 (63)		1 CRC (58)	
	9			1 (21)			2 (45, 45)					
	10			2 (2,15)			1 (45)				1 EC (16) 1 CRC (26)	1 STS+EC+CRC
	11			2 (13,38)			1 (35)					1 STS+BC
	12			2 (54,68)								
	13				1 (7)		1 (28) 1 bilateral (35,39)	1 (54)			1 kidney	1 CM
	14			1 (U)			2 (U)	1 GBM (U)			1 stomach, 1 ovarian 1 NHL	1 BC+STS
	15		1 breast (22)				2 (43,50)				1 head&neck (45) 1CRC (70)	
	16		1 chest wall (56)				1 bilateral (48,61) 1 (80)				1 peritoneum (58)	1 CM+AS
	17		1 breast (32)								1 ALL (15)	1 ALL+BA
	18		1 breast (57)				2 (49,58)				1 prostate (51)	1 CM+BA
	19		2 bilateral breast (37,39)				1 (50)			1 (60)		
	20		1 breast (42)				1 bilateral (37,45) 1 (64)					1 CM+BA+BC
	21		1 thigh (17)	1 (U)								
	22		1 breast (15)									

ALL, acute lymphoblastic leukemia; AS, angiosarcoma; astroc., astrocytic tumour; BA, breast angiosarcoma; BC, breast cancer; CAS, cardiac angiosarcoma; CM, cutaneous melanoma; CNS, central nervous system; CRC, colorectal cancer; EC, endometrial cancer; GBM, glioblastoma multiforme; NHL, non-Hodgkin Lymphoma; NS, not specified; NT, not tested; PTC, papillary thyroid carcinoma; STS, soft tissue sarcoma; U, unknown.

Tumours of the members of family 1 without DNA sample were NT. Age at diagnosis is shown in brackets.

^*^Multiple primary tumours refer to tumour types described in one individual.

**Table 2:** Deleterious germinal mutations described in the *POT1* gene.

**Position**	**Allele**	**Exon**	**Amino acid change**	**Domain**	**Source**
g.124503601	G>A	4	p.Arg117Cys	OB1	*
g.124503684	T>C	4	p.Tyr89Cys	OB1	Robles-Espinoza *et al*.^20^
g.124503670	G>C	4	p.Gln94Glu	OB1	
g.124493077	C>A	6	p.Arg273Leu	OB2	
g.124465412	C>T	14	Splice site	Telomere/TPP1 binding	
g.124503540	C>T	4	p.Arg137His	OB1	Shi *et al*.^21^†
g.124499043	C>T	5	p.Asp224Asn	OB2	
g.124493086	C>T	6	p.Ser270Asn	OB2	
g.124469308	C>G	13	p.Ala532Pro	Telomere/TPP1 binding	
g.124464052	C>G	15	p.Gln623His	Telomere/TPP1 binding	
g.124503667	C>A	4	p.Gly95Cys	OB1	
g.124481048	C>A	10	p.Glu450‡	Telomere/TPP1 binding	Bainbridge *et al*.^22^
g.124464068	TTA>T	15	p.Asp617Glu (fs)	Telomere/TPP1 binding	

fs, frame shift; POT1, protection of telomeres 1.

^*^Mutation described in the present study.

^†^Founder mutation.

^‡^Stop codon.
