# Supplementary material for: A mutation in the POT1 gene is responsible for cardiac angiosarcoma in TP53-negative Li–Fraumeni-like families
Source: Nat Commun. 2015 Sep 25;6:8383. doi: 10.1038/ncomms9383 (PMC4598567; doi:10.1038/ncomms9383)
Supplement: Supplementary Information — Supplementary Figures 1 and 2 and Supplementary Tables 1-6 [file ncomms9383-s1.pdf]

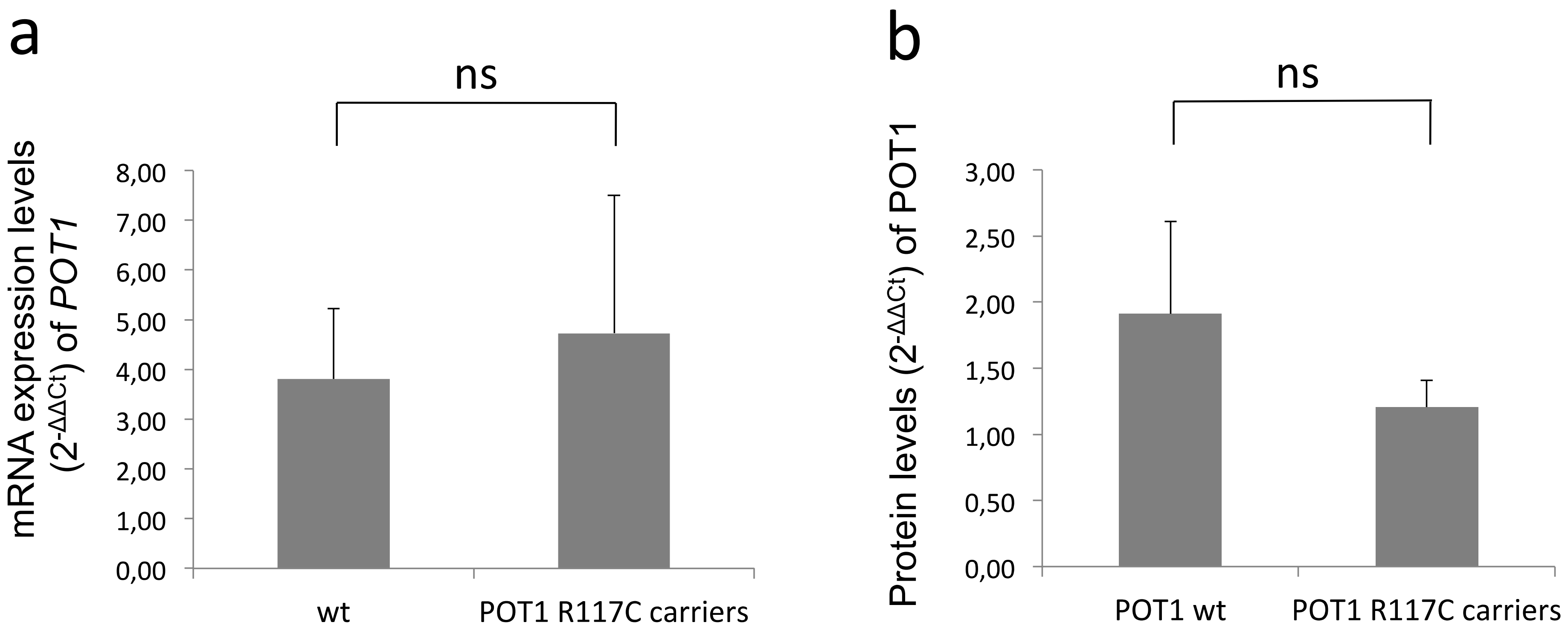

Supplementary Figure 1: Analyses of family 1 members. The T-student's t –test was used for the statistical analysis of normal distributed values. a) Comparison of mRNA expression levels between *POT1* wild type (wt) and p.R117C mutation carriers. normalized with *GAPDH* as control b) Values of POT1 protein levels for several members of family 1 (n=7) normalized for GAPDH control . There was a trend towards decreased POT1 levels in mutation carriers (1.91±0.69) compared with non-carriers (1.21±0.2) (p=0.1).

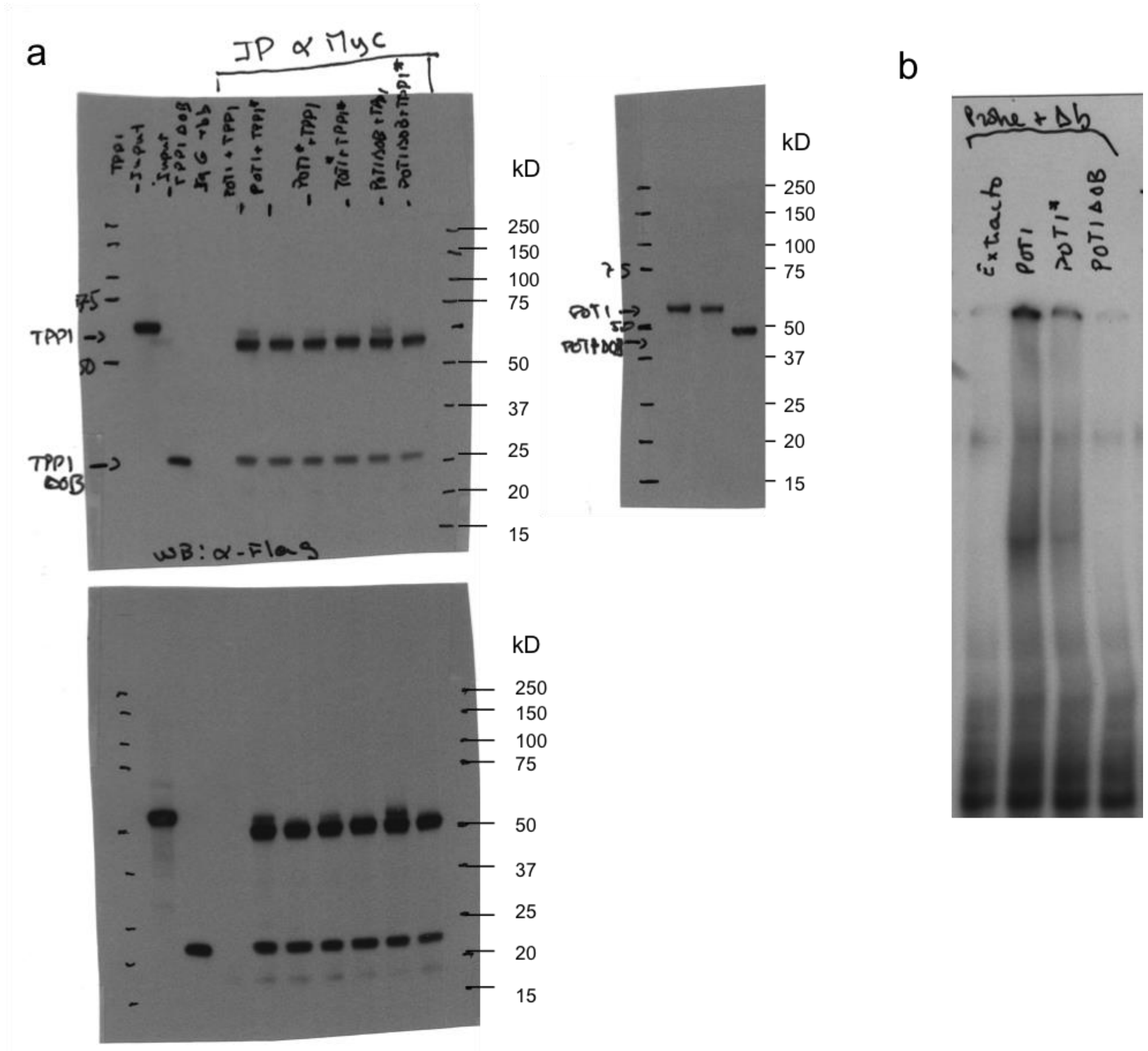

Supplementary Figure 2: Uncropped scans of the gels/blots. (a) Western blots presented in Figure 3d and (b) of the electrophoretic mobility shift assay (EMSA) presented in Figure 3e. The molecular weight ladder is shown to the right of each blot.

Supplementary Table 1: Candidate variants to study further segregation in members of family 1.

Prioritization Criteria	Variant position	GENE	Protein change	Function	Tissue expression	COSMIC/Pathology/
Described in COSMIC	chr1:1238583	*ACAP3*	Arg62Cys	arf-GAP with coiled-coil, ANK repeat and PH domain-containing protein 3	Lung	COSM527726
	chr15:71535077	*THSD4*	Thr185Met	A disintegrin and metalloproteinase with thrombospondin motifs-like protein 6	Lung	COSM555837
	chr7:5112796	*RBAK*	Leu227Phe	RB-associated KRAB zinc finger protein		pleomorphic sarcoma (RB1)
Function Related	chr7:124503601	*POT1*	Arg117Cys	Telomere end-binding protein	Blood/ nervous	Gastric carcinoma, melanoma, glioma
	chr2:37347280	*EIF2AK*	Ser316*	Apoptosis		*p53* related
	chr7:151945007	*MLL3*	Gly838Ser	Lysine N-methyltransferase 2C	Muscular	myeloid/lymphoid or mixed-lineage leukemia
Tissue Related	chr5:79030433	*CYMA5*	His1949Tyr		Cardiac	
	chr2:220355477	*SPEG*	Val3062Leu	Differentiation	Aortic	
	chr1:161721670	*DUSP12*	Phe158Ser	Protein-tyrosine phosphatase		
	chr10:81932595	*ANXA11*	Phe8Leu	Annexin		

*Stop codon

Supplementary Table 2: Haplotype frequency study for families 1and 2.

SNP	Position	Annotation	MAF	1*	2	3	4	5	6
rs67585224	124537476	Intron 1	0.21	A	C	A	A	A	C
rs68091803	124526331	Intron 2	0.20	T	T	T	T	T	T
rs7784168	124492038	Intron 6	0.28	C	C	T	T	T	T
rs7801661	124483222	Intron 8	0.21	T	C	T	T	T	T
rs35536751	124481185	Exon10	0.01	C	C	C	C	C	C
rs17246404	124462661	3'UTR	0.20	C	T	C	T	C	T
rs76436625	124462655	3UTR	0.12	T	T	T	T	C	T
Frequency				42.0%	21.1%	7.7%	7.7%	3.3%	2.2%

* Unique haplotype found in different members of families 1 and 2.

MAF: Minor Allele Frequency.

Supplementary Table 3: In silico PACC score of aa annotated in the OB fold and described stacking to DNA (T1-G10) [14] for POT1 proteins with mutations within OB1 domain (Categorization is shown in brackets).

Interaction	Postion (aa)	WT	p.Y89>C	p.Q94>G	p.G95>C	p.R117>C	p.R137>H	OBDelta*	OBDelta**
OB fold	146	122(E)	122(E)	122(E)	122(E)	122(E)	122(E)	122(E)	97(E)
	147	93(E)	117(E)	93(E)	93(E)	93(E)	117(E)	117(E)	93(E)
	148	45(I)	45(I)	45(I)	45(I)	45(I)	45(I)	95(E)	45(I)
	149	59(E)	59(E)	79(E)	59(E)	59(E)	59(E)	59(E)	59(E)
	150	19(I)	9(B)	19(I)	9(B)	9(B)	9(B)	9(B)	9(B)
	151	32(I)	32(I)	32(I)	32(I)	61(I)	9(B)	19(I)	49(I)
	152	86(E)	86(E)	86(E)	86(E)	0(B)	61(I)	86(E)	86(E)
T1; T2	62	0(B)	0(B)	0(B)	0(B)	0(B)	0(B)	-	-
A3; G4	89	26(I)	16(I)	26(I)	26(I)	26(I)	26(I)	-	-
G5; G6	31	0(B)	0(B)	0(B)	0(B)	0(B)	0(B)	-	-
T7	271	26(I)	26(I)	26(I)	26(I)	26(I)	26(I)	66(I)	66(I)
	161	0(B)	0(B)	0(B)	0(B)	0(B)	0(B)	0(B)	0(B)
	245	0(B)	0(B)	0(B)	0(B)	0(B)	0(B)	0(B)	0(B)
T8; A9	266	36(I)	0(B)	36(I)	22(I)	0(B)	0(B)	55(I)	22(I)
G10	223	0(B)	0(B)	0(B)	0(B)	0(B)	0(B)	0(B)	0(B)

*: 1-126 deletion; **: 10-141 deletion; (E): Exposed; (I): Intermediate; (B): Buried

Highlighted in grey: Most significant changes

Supplementary Table 4: In silico protein-protein interaction score for positions with significant acceptance in wt POT1 (threshold >20) for POT1 proteins with mutations within OB1 domain.

Position (aa)	WT	p.Y89>C	p.Q94>G	p.G95>C	p.R117>C	p.R137>H	OBDelta*	OBDelta**
1 (M)	26	26	26	26	28	24	NA	19
2 (S)	30	31	26	30	27	24	NA	10
3 (L)	27	25	25	28	25	21	NA	-23
39 (K)	26	24	24	24	24	22	NA	NA
91 (K)	26	29	29	28	31	29	NA	NA
253 (S)	33	28	35	34	30	36	38	32
254 (E)	28	19	24	25	31	31	21	28
255 (N)	34	23	26	28	36	36	29	38
341 (Q)	23	23	15	13	21	28	23	19
363 (R)	47	48	47	48	47	49	49	42
427 (K)	23	21	23	29	20	26	10	10
428 (N)	23	17	20	23	17	22	10	6
499 (H)	21	19	16	20	-24	15	18	-23
599 (A)	28	23	17	23	24	28	23	19

*: 1-126 deletion; **: 10-141 deletion; NA: Not applicable

Highlighted in grey: Most significant changes

Supplementary Table 5: Primers used for variant validation and further segregation and for *POT1* gene and haplotype studies.

Gene	Position of the variant	Forward	Reverse	TºC	Product size (bp)
ACAP3	chr1:1238583	GCTGGTGAAGCTGTGCAGT	GTAGGCTGTCAGCGAACCTC	57	338
THSD4	chr15:71535077	CCCTGGCAAGTATGGCTATG	ACTTGAAGCTGCAGGGTAGC	64	286
RBAK	chr7:5112796	TTCTAGCCAGCACCAGGAAG	CCCAAAAGGAAACAGCACCT	64	364
POT1	chr7:124503601	AATGATGCTCCGTCCACTTC	TGGTTCGTAGGTTGTGCATC	64	351
EIF2AK	chr2:37347280	ATGTTGGCCAGACTGGTTTC	CCGGAGTAGCTGGGACTACA	64	609
MLL3	chr7:151945007	TCAGTCACTCCAAAAATTGG	TGGCATTCTTCATTAACAGCA	65	260
CYMA5	chr5:79030433	GATGTTGGGAAAGCCAGAAA	GAATCTCCTTCCCCTCTGCT	59	350
SPEG	chr2:220355477	GCCACACTCCAGGGTTATTG	AAGATCGTGCCCTATGCTG	64	245
DUSP12	chr1:161721670	TGCAGGAGTCAGTCGAAGTG	TCTCAGGGATTGCTGGAAGT	64	405
ANXA11	chr10:81932595	GAGGAGGGGAGTGAGGAAAC	CACCAGCAGGAGACAGTGAA	64	326

Gene	EXON	Forward	Reverse	TºC	Product size (bp)
POT1	1	TTAGGCTGGAGATGGGTAAAA	CACATGTATCTATGTGTGTGGCATA	60	589
	2.1	CATGGATTTGCTGCTAATATGAT	AATGCATTTCCACTCCAAAAA	58	246
	2.2	AATTGTACCATTTATAAACAAAGTTC	GCATCAGTGTTGTTTGGCAAT	58	451
	3	TGCTTTAAAATATTGAAAGTCAGG	CAGTGAACAATACAGAGTTCTCTTCAA	58	238
	4	TGGTTCGTAGGTTGTGCATC	GGTGCTAACTTATAATTCCCAGTA	58	400
	5	TTTTTCAAAAGGATCATAAAACTACTC	AAAAATTGCTCTAACCCATTAAG	58	398
	6	CCACTTTCTAAATAACAAATGATCT	TCGGCTTAATCGATACCTTA	58	250
	7	TCTGGATTTTGTGGGTAGAGC	AGCAAGAACTAAACTGTCAATGTT	58	199
	8	AGCAACGCTCCATTGTTTTC	TGCTCATTACTGTGCCCATC	58	175
	9	GCCTAGCGTAATATTTTCTTAGC	CGTTTGTTTTATTATGGAAAATACTCA	60	500
	10	TCACGCTTACACCAAAATCG	GCAAAAGGAGTATTCTAACAAAACA	58	300
	11	CATTTTATTTGCACTACTTGAAGG	AGCACATGACCCCAGAACTT	60	248
	12	GCAGTAAAAAGTTATAACAAACAAGC	TCTGAATTGGCCAAACAACAT	60	494
	13	GCAGACTCATATGGTTTGATCTTTT	TCCTTTTAGGAACAAAGCAGGT	60	224
	14	TTTTGGAGTTGAGACCAGCA	GGGATTGTTAAAATATTCTTGCCTAC	60	298
	15	CAGAGTTATTTATTTTGTTTTAATGG	AAACTCAGGTCAGGAAAAGA	60	250

Gene	Position of the SNP	Forward	Reverse	TºC	Product size (bp)
POT1	rs67585224	TCCTGAGAACTGTCCTGAGAACT	AAAATATTCAAATTCACTAGGATGACA	57	205
	rs68091803	AGCCATGGGGAACAAGAATA	TTTTCACGAAAAATTCTAGCTAATGA	57	586
	rs7784168	TCTGGATTTTGTGGGTAGAGC	AGCAAGAACTAAACTGTCAATGTT	58	199
	rs7801661	GCCTAGCGTAATATTTTCTTAGC	CGTTTGTTTTATTATGGAAAATACTCA	62	500
	rs35536751	TCACGCTTACACCAAAATCG	GCAAAAGGAGTATTCTAACAAAACA	58	300
	rs17246404<br>rs76436625	AGAATATAGACTCTTGGTTCAAAACAT	CTTCTAGATTGAGGGCTTCCTG	57	150

.

Supplementary Table 6: Characteristics of the antibodies used for Western blotting and FISH studies.

Antibody	Reference	Clone	Working Concentrations	
			WB	IF
POT1*	Novus Biologicals: NB500-176	Rabbit Polyclonal	1:500	1:250
GAPDH	Homemade, CNIO	Monoclonal	1:500	-
TRF2*	Cell Signaling, #2645	Rabbit Polyclonal	-	-
TRF1	Homemade, CNIO	Rabbit Polyclonal	-	1:250
TRF1*	(TRF-78) (ab10579), ABCAM	Mouse monoclonal	-	1:250
γH2AX	Millipore, #05-636	Mouse Monoclonal	-	1:250
555-Alexa-donkey	Life technologies, A31572		-	1:400
488-Alexa-goat	Life technologies, A11017		-	1:400
FLAG	F3166 SIGMA	Mouse monoclonal	1:1000	-
c-MYC (9E10)	C-40, Santa Cruz Biotechnology	Mouse monoclonal	1:1000	1:500

*: 4 μL of antibody were used per IP Chip reaction; WB: Western Blot; IF: Inmunofluorescence
